# Transcriptional and post‐transcriptional regulation of heading date in rice

**DOI:** 10.1111/nph.17158

**Published:** 2021-02-14

**Authors:** Shirong Zhou, Shanshan Zhu, Song Cui, Haigang Hou, Haoqin Wu, Benyuan Hao, Liang Cai, Zhuang Xu, Linglong Liu, Ling Jiang, Haiyang Wang, Jianmin Wan

**Affiliations:** ^1^ State Key Laboratory for Crop Genetics and Germplasm Enhancement Jiangsu Plant Gene Engineering Research Center Nanjing Agricultural University Nanjing 210095 China; ^2^ National Key Facility for Crop Gene Resources and Genetic Improvement Institute of Crop Science Chinese Academy of Agricultural Sciences Beijing 100081 China

**Keywords:** expansion of rice areas, flowering, genetic pathways, heterosis, molecular breeding, *Oryza sativa*

## Abstract

Rice is a facultative short day (SD) plant. In addition to serving as a model plant for molecular genetic studies of monocots, rice is a staple crop for about half of the world’s population. Heading date is a critical agronomic trait, and many genes controlling heading date have been cloned over the last 2 decades. The mechanism of flowering in rice from recognition of day length by leaves to floral activation in the shoot apical meristem has been extensively studied. In this review, we summarise current progress on transcriptional and post‐transcriptional regulation of heading date in rice, with emphasis on post‐translational modifications of key regulators, including Heading date 1 (Hd1), Early heading date 1 (Ehd1), Grain number, plant height, and heading date7 (Ghd7). The contribution of heading date genes to heterosis and the expansion of rice cultivation areas from low‐latitude to high‐latitude regions are also discussed. To overcome the limitations of diverse genetic backgrounds used in heading date studies and to gain a clearer understanding of flowering in rice, we propose a systematic collection of genetic resources in a common genetic background. Strategies in breeding adapted cultivars by rational design are also discussed.

## Introduction

Following seed germination rice undergoes a vegetative growth phase before panicle initiation, at which there is a change in apical meristem identity from a vegetative meristem to a floral meristem as the plant enters the reproductive phase that leads to flowering. Flowering time (also known as heading date) is an important agronomic trait that determines seasonal and regional adaptation. If a cultivar flowers too early in a specific location there will be inadequate use of light and temperature resources, and consequent lower yield. By contrast, if a cultivar is too late in heading it cannot complete flowering and grain development before the onset of cold, also resulting in lower yield.

Heading date in rice is affected by exogenous factors such as photoperiod, temperature and nutrient availability (e.g. nitrogen levels), among which photoperiod is a key factor. Rice is a facultative short day (SD) plant; flowering is promoted under SD conditions and inhibited by long day (LD) conditions. However, in the juvenile growth stage of the vegetative growth phase, flowering cannot be promoted even under inductive daylength conditions. This stage is called the basic vegetative phase (BVP). Only after completion of the BVP can rice respond to photoperiodic stimuli for flowering, and this stage is called the photoperiod‐sensitive phase (Vergara & Chang, 1985; Nishida *et al*., 2001). The length of BVP and the sensitivity to exogenous factors is determined by endogenous genetic composition, a fundamental factor that determines differences in photoperiod response among rice genotypes and leads to extensive variation in heading date.

## Genetic pathways controlling flowering in rice

The general process of photoperiod‐induced flowering is conserved in LD and SD plants. First, the leaves measure the photoperiod and generate a mobile flowering signal called a florigen under inductive daylength. The florigen is then transported from leaves to the shoot apex where the shoot apical meristem (SAM) perceives the florigen signal and activates downstream floral identity genes to trigger transition to flowering. The genetic pathway for photoperiod‐controlled flowering has been intensively studied in the model plant Arabidopsis, a facultative LD plant. In this plant, GIGANTEA (GI) protein promotes *CONSTANS* (*CO*) transcription in leaves, and CO protein directly activates transcription of the florigen *FLOWERING* *LOCUS* *T* (*FT*). However, there are multiple additional layers of transcriptional and post‐transcriptional modifications that regulate the photoperiodic flowering pathway to influence flowering time (Blumel *et* *al*., 2015).

**Fig. 1:**
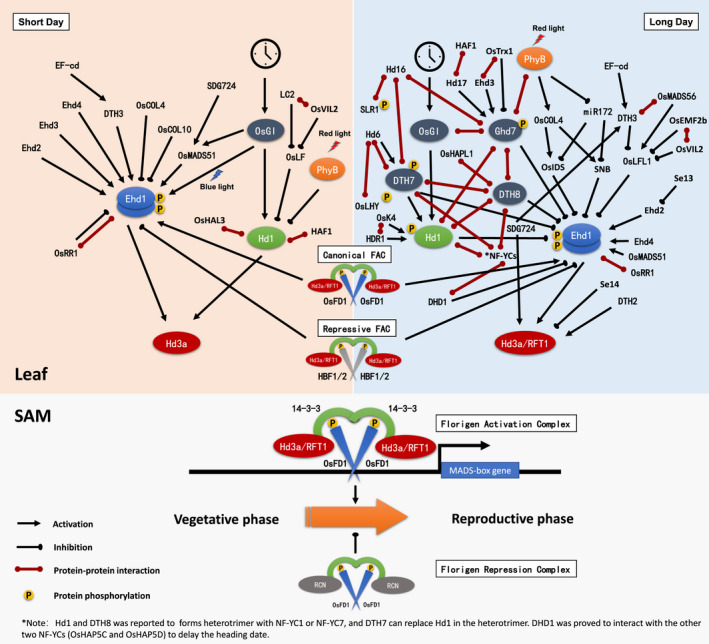
Genetic pathways controlling heading date in rice. Schematic representation of genetic pathways controlling flowering in rice. A circadian clock is shown at the top. Key heading date genes are indicated in oval backgrounds. Arrows indicate upregulation, and bars indicate downregulation. Rice has two florigen genes, *Hd3a* and *RFT1*, short day (SD) conditions accelerate heading by promoting the expression of *Hd3a* through *Hd1* and *Ehd1*. Expression of *Ehd1* can be upregulated or downregulated by many genes. *Hd1* can be activated by *OsGI* under SD conditions. In long day (LD) conditions, *Hd1* function reverses to flowering inhibition; this reversal is controlled by many other factors such as *Ghd7* and *DTH8* and is mediated by protein–protein interactions. The florigen proteins Hd3a and RFT1 are transported to the shoot apical meristem (SAM) after induction in the leaves. In the SAM, they form the florigen activation complex (FAC), and activate the expression of downstream targets, including members of MADS‐box transcription factor family genes that control phase transition of the SAM from vegetative to reproductive development. FAC and repressive FAC‐like complexes can also be formed in the SAM and leaves to fine tune the heading date.

Using forward and reverse genetics, as well as population genetic studies, many genes involved in photoperiodic flowering have been cloned into rice (Table 1). Most of these genes function upstream of the florigen genes. These genes can be roughly grouped to two signalling pathways with some crosstalk. The evolutionarily conserved *OsGI–Hd1–Hd3a* pathway (analogous to the Arabidopsis *GI*–*CO*–*FT* pathway) and *Ehd1*‐centred specific pathway in monocots involve *Ehd1*, *Ghd7*, *Early* *heading* *date* *2* (*Ehd2*, also known as *RID1*, or *OsId1*), *Early* *heading* *date* *3* (*Ehd3*), and *Early* *heading* *date* *4* (*Ehd4*), which has no counterpart in Arabidopsis (Fig. 1). Both pathways are finally integrated to florigen. Unlike Arabidopsis that has only one florigen gene, rice has two florigen genes, *Heading* *date* *3a* (*Hd3a*) and *RICE* *FLOWERING* *LOCUS* *T* *1* (*RFT1*). Both genes encode a phosphatidylethanolamine binding protein that is a close homologue of Arabidopsis FT.

**Table 1:** Cloned genes controlling heading date in rice.

Gene symbol	Effect	Locus ID	Description	Reference
*OsGI*	SD/LD promotion	Os01g0182600	Encodes an orthologue of Arabidopsis GIGANTEA, a plant‐specific nuclear protein that functions in diverse physiological processes	Hayama *et al*. (2002)
*OsEF3*	SD/LD promotion	Os01g0566100	Encodes an orthologue of Arabidopsis ELF3, a putative nematode responsive protein‐like protein	Fu *et al*. (2009)
*OsLFL1*	LD repression	Os01g0713600	Encodes a putative B3 DNA‐binding domain‐containing transcription factor	Peng *et al*. (2008)
*OsWOX13*	LD promotion	Os01g0818400	Encodes a WUSCHEL homeobox transcription factor	Minh‐Thu *et al*. (2018)
*OsHESO1*	Maybe repression	Os01g0846450	Encodes a homologue of *Arabidopsis thaliana* HEN1 suppressor 1	Yano *et al*. (2016)
*OsMADS51*	SD promotion	Os01g0922800	Encodes a type I MADS‐box transcription factor	Kim *et al*. (2007)
*SE13*	LD promotion	Os01g0949400	Encodes phytochromobilin synthase, an orthologue of Arabidopsis HY2, a key enzyme in phytochrome chromophore biosynthesis	Saito *et al*. (2011)
*LC2*	SD promotion	Os02g0152500	Encodes a vernalisation insensitive 3‐like protein, a possible component of the PRC2 complex mediating H3K27me3 in target genes	Wang *et al*. (2013)
*OsUbDKγ4*	LD repression	Os02g0290500	Encodes ubiquitin‐like domain kinase γ4	Song *et al*. (2017)
*RCN2*	SD/LD repression	Os02g0531600	Encodes rice TFL1‐like proteins that form a florigen repression complex (FRC) with 14‐3‐3 and OsFD1	Nakagawa *et al*. (2002), Kaneko‐Suzuki *et al*. (2018)
*SDG725*	SD/LD promotion	Os02g0554000	Encodes histone H3 lysine 36‐specific methyltransferase	Sui *et al*. (2013)
*OsCOL4*	SD/LD repression	Os02g0610500	Encodes a member of the rice CONSTANS‐like (COL) family protein	Lee *et al*. (2010)
*DTH2*	LD promotion	Os02g0724000	Encodes a CONSTANS‐like protein that probably acts independently of Hd1 and Ehd1	Wu *et al*. (2013)
*HDR1*	LD repression	Os02g0793900	Encodes a homologue of the ligand of the SNF1/AMPK/SnRK1 kinase PpSKI in moss	Sun *et al*. (2016)
*Ehd4*	SD/LD promotion	Os03g0112700	Encodes an *Oryza*‐genus‐specific CCCH‐type zinc finger protein	Gao *et al*. (2013)
*Ef‐cd*	SD/LD promotion	Os03g0122500	A long noncoding RNA transcribed from the antisense strand of the DTH3 locus, and positively regulates expression of DTH3	Fang *et al*. (2019)
*DTH3*	SD/LD promotion	Os03g0122600	Encodes a typical MIKC‐type MADS‐box protein OsMADS50, the putative SOC1/AGL20 orthologue in rice	Lee *et al*. (2004), Bian *et al*. (2011)
*SE14*	LD repression	Os03g0151300	Encodes a JmjC domain‐containing protein that functions in demethylation of H3K4me3 in *RFT1*	Yokoo *et al*. (2014)
*OsDof12*	LD promotion	Os03g0169600	Encodes a DNA‐binding one finger (Dof) protein	Li *et al*. (2009)
*OsNF‐YC2*	LD repression	Os03g0251350	Encodes the HAP5 subunit of the heme activator protein (HAP) complex	Kim *et al*. (2016)
*SDG718*	SD promotion	Os03g0307800	Encodes enhancer of zeste 1, a key component of the PRC2 complex	Liu *et al*. (2014)
*PHYB*	LD repression	Os03g0309200	Encodes photoreceptor phytochrome B	Takano *et al*. (2005)
*OsCOL10*	SD/LD repression	Os03g0711100	Encodes a member of rice CONSTANS‐like (COL) family proteins	Tan *et al*. (2016)
*PHYA*	LD repression	Os03g0719800	Encodes photoreceptor phytochrome A	Takano *et al*. (2005)
*OsWDR5*	SD/LD promotion	Os03g0725400	Encodes WD repeat‐containing protein 5	Jiang *et al*. (2018)
*PHYC*	LD repression	Os03g0752100	Encodes photoreceptor phytochrome C	Takano *et al*. (2005)
*Hd6*	LD repression	Os03g0762000	Encodes the alpha subunit of protein kinase CK2	Takahashi *et al*. (2001)
*Hd16*	LD repression	Os03g0793500	Encodes a casein kinase‐I protein	Dai & Xue (2010), Hori *et al*. (2013)
*OsSFL1*	SD promotion	Os04g0166600	Encodes a rice homologue of yeast SAP30, a component of histone deacetylation	Geng *et al*. (2020)
*SDG708*	SD/LD promotion	Os04g0429100	Encodes SET DOMAIN GROUP 708 protein, a histone H3 lysine 36‐specific methyltransferase	Liu *et al*. (2016)
*OsRR1*	SD repression	Os04g0442300	Encodes the type‐A response regulator that forms a heterodimer with Ehd1	Cho *et al*. (2016)
*OsCRY1b*	SD/LD promotion	Os04g0452100	Encodes cryptochrome, the blue/UV‐A light photoreceptor	Hirose *et al*. (2006)
*RFL*	promotion	Os04g0598300	Encodes the rice homologue of transcription factor LFY	Rao *et al*. (2008)
*HAF1*	SD/LD promotion	Os04g0648800	Encodes a RING‐finger ubiquitin ligase	Yang *et al*. (2015)
*OsHAPL1*	LD repression	Os05g0494100	Encodes Heme activator protein like 1	Zhu *et al*. (2017)
*OsLF*	SD/LD repression	Os05g0541400	Encodes an atypical HLH protein	Zhao *et al*. (2011)
*Hd17*	SD/LD promotion	Os06g0142600	Encodes a homologue of Arabidopsis ELF3 protein	Matsubara *et al*. (2012), Saito *et al*. (2012)
*RFT1*	LD promotion	Os06g0157500	Encodes a rice florigen	Komiya *et al*. (2008)
*Hd3a*	SD promotion	Os06g0157700	Encodes a florigen that is an orthologue of Arabidopsis FT	Kojima *et al*. (2002)
*OsHAL3*	SD promotion	Os06g0199500	Encodes a flavin mononucleotide‐binding protein	Sun *et al*. (2009)
*Hd1*	SD promotion/LD repression	Os06g0275000	Encodes the homologue of Arabidopsis CONSTANS	Yano *et al*. (2000)
*SDG711*	LD repression	Os06g0275500	Encodes enhancer of zeste 1, a key component of the PRC2 complex	Liu *et al*. (2014)
*SE5*	SD/LD repression	Os06g0603000	Encodes an enzyme implicated in phytochrome chromophore biosynthesis	Andrés *et al*. (2009)
*OsFTIP1*	SD/LD promotion	Os06g0614000	Encodes FT‐INTERACTING PROTEIN1	Song *et al*. (2017)
*OsNF‐YC4*	LD repression	Os06g0667100	Encode the HAP5 subunit of the heme activator protein (HAP) complex	Kim *et al*. (2016)
*OsMADS15*	promotion	Os07g0108900	Encodes a MADS‐box transcription factor	Lu *et al*. (2012)
*Ghd7*	LD repression	Os07g0261200	Encodes a CCT domain protein	Xue *et al*. (2008)
*OsCOL13*	SD/LD repression	Os07g0667300	Encodes a CONSTANS‐like transcriptional activator	Sheng *et al*. (2016)
*DTH7*	LD repression	Os07g0695100	Encodes pseudoresponse regulator protein OsPRR37	Koo *et al*. (2013), Yan *et al*. (2013), Gao *et al*. (2014)
*Ehd3*	LD promotion	Os08g0105000	Encodes a plant homeodomain finger‐containing protein	Matsubara *et al*. (2011)
*Hd18*	SD/LD promotion	Os08g0143400	Encodes a histone acetylase related to Arabidopsis FLOWERING LOCUS D	Shibaya *et al*. (2016)
*DTH8*	SD promotion/LD repression	Os08g0174500	Encodes the AP3 subunit of heme activator protein (HAP) complex	Wei *et al*. (2010), Yan *et al*. (2011)
*SDG701*	SD/LD promotion	Os08g0180100	Encodes the SET domain group protein	Liu *et al*. (2017)
*GF14c*	promotion	Os08g0430500	Encodes a G‐box factor homologue of 14‐3‐3	Taoka *et al*. (2011)
*OsK4*	LD repression	Os08g0484600	Encodes snf1‐related kinase interactor 2 that interacts with HDR1 and phosphorylates Hd1	Sun *et al*. (2016)
*OsNF‐YC6*	SD/LD repression	Os08g0496500	Encodes a HAP5 subunit of the heme activator protein (HAP) complex	H. Zhang *et al*. (2019)
*OsCOL15*	SD/LD repression	Os08g0536300	Encodes a rice CONSTANS‐like protein	Wu *et al*. (2018)
*SDG723*	LD promotion	Os09g0134500	Encodes a trithorax group chromatin‐remodelling factor, an homologue of Arabidopsis Trithorax protein1 (ATX1)	Choi *et al*. (2014)
*OsCO3*	SD repression	Os09g0240200	Encodes a CONSTANS‐LIKE protein	Kim *et al*. (2008)
*OsEMF2b*	SD/LD promotion	Os09g0306800	Encodes a polycomb‐group (PcG) protein, a homologue of Arabidopsis EMF2	Yang *et al*. (2013), Xie *et al*. (2015)
*SDG724*	SD/LD promotion	Os09g0307800	Encodes histone methyltransferase	Sun *et al*. (2012)
*OsMADS8*	SD/LD promotion	Os09g0507200	Encodes a MADS‐box transcription factor	Kang *et al*. (1997)
*OsFD1*	promotion	Os09g0540800	Encoding a bZIP transcription factor	Taoka *et al*. (2011), Tsuji *et al*. (2013)
*Ehd2*	SD/LD promotion	Os10g0419200	Encodes the Cys2/His2‐type zinc finger transcription factor, an orthologue of maize INDETERMINATE1	Matsubara *et al*. (2008), Wu *et al*. (2008)
*Ehd1*	SD/LD promotion	Os10g0463400	Encodes a B‐type response regulator	Doi *et al*. (2004)
*OsMADS56*	LD repression	Os10g0536100	Encodes a MADS‐box protein	Ryu *et al*. (2009)
*RCN1*	SD/LD repression	Os11g0152500	Encodes FL1‐like proteins that form a florigen repression complex (FRC) with 14‐3‐3 and OsFD1	Nakagawa *et al*. (2002), Kaneko‐Suzuki *et al*. (2018)
*Unnamed gene*	Maybe repression	Os11g0187200	Encodes a GATA zinc finger‐type transcription factor that was identified in a GWAS study	Yano *et al*. (2016)
*OsFKF1*	SD/LD promotion	Os11g0547000	Encodes a homologue of FLAVIN‐BINDING, KELCH REPEAT, F‐BOX 1 (FKF1)	Han *et al*. (2015)
*DHD1*	SD/LD repression	Os11g0706200	Encodes a GRAS family protein	H. Zhang *et al*. (2019)
*OsVIL2*	SD/LD promotion	Os12g0533500	Encodes a vernalisation insensitive 2‐like protein, a possible component of the PRC2 complex mediating H3K27me3 in target genes	Wang *et al*. (2013), Yang *et al*. (2013)

### Recognition of SDs through circadian clock and light signals

**Fig. 2:**
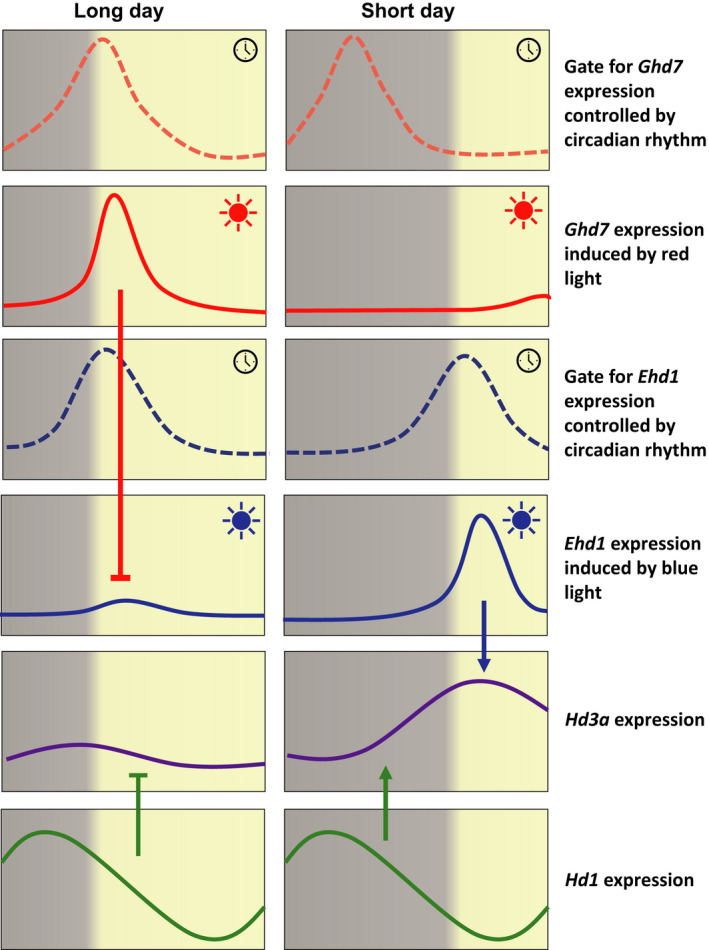
External coincidence model of rice heading regulation by circadian clock and day length. Diurnal expression of floral regulators Ghd7, Ehd1, Hd1 and Hd3a under long days (LD) and short days (SD). Arrows indicate induction, and bars indicate suppression of gene expression. *Ghd7* expression has the highest red‐light inducibility around dawn under LD, whereas the inducibility peak shifts to midnight under SD conditions. *Ehd1* expression has the highest blue‐light inducibility around dawn under both LD and SD conditions. The light‐sensitive phase set by the circadian clock is called a gate, which is indicated by dashed curves. Under LD, red light induces *Ghd7* transcription around dawn, further suppressing the expression of *Ehd1* and *Hd3a*. However, under SD, *Ehd1* expresses around dawn and induces expression of *Hd3a*. *Hd1* expression shows a diurnal rhythm and reaches a peak during the night under both SD and LD, but under LD, mRNA is also produced for several hours during the light phase. Dark phase‐expressed *Hd1* promotes flowering under SD, whereas light phase‐expressed *Hd1* inhibits flowering under LD.

As a SD plant, flowering in rice is induced when the day length becomes less than a specific threshold, known as the critical day length. When the day length is shorter than 13 h, the florigen gene *Hd3a* is expressed in the morning even in rice seedlings, and when day length exceeds 13.5 h, expression of *Hd3a* decreases to less than one‐tenth of the 13 h d length level (Itoh *et* *al*., 2010). Based on physiological studies, it has been hypothesised that plants monitor day length changes by recognising light conditions at a specific time of day (Bünning, 1960). In rice, coincidence between a circadian clock controlled fluctuating internal signal and a periodic external signal can start the flowering process, and this mechanism is known as external coincidence (Song *et* *al*., 2015). Many heading date genes show rhythmic expression that is dependent on day length. *OsGI* has a rhythmic expression pattern that peaks at the end of the light period and activates expression of *Hd1*. *Hd1* encodes the homologue of Arabidopsis CO and shows diurnal rhythmic expression that reaches a peak during the night both under SD and LD conditions but, under LD conditions, mRNA is also produced for several hours during the light phase (Izawa *et* *al*., 2002). Although *Hd1* promotes flowering under SD, artificial overexpression of *Hd1* during the light phase inhibits flowering under SD conditions (Fig. 2). This suggests that exposure of *Hd1* to light converts it to a repressor of flowering (Ishikawa *et* *al*., 2011). Night‐break treatment under SD by exposure of rice plants to as little as 10 min of light in the middle of a long night results in delayed flowering and failure to induce *Hd3a* expression (Ishikawa *et* *al*., 2005).

*Ghd7* encodes a CCT domain‐containing protein and is a key floral repressor in rice under LD conditions (Xue *et* *al*., 2008; Cai *et* *al*., 2019). *Ghd7* is expressed in the morning under LD conditions. This day length‐dependent expression is achieved by a complex gating mechanism. A red‐light signal mediated by phytochromes induces *Ghd7* expression only at a specific time of day when the gate (a light‐sensitive phase set by the circadian clock) for *Ghd7* expression is open. Under LD conditions, the *Ghd7* gate is set to open around dawn, and light induces *Ghd7* expression. Under SD conditions it opens at midnight, therefore there is no light to induce *Ghd7* expression (Itoh *et* *al*., 2010). By contrast with the *Ghd7* gate for red light, the *Ehd1* gate for blue light is not affected by day length. *Ehd1* expression can be induced by blue‐light treatment in the morning in an *OsGI*‐dependent manner under both LD and SD conditions. Because *Ehd1* expression is repressed by *Ghd7*, red light induces *Ghd7* transcription, leading to suppression of *Ehd1* and *Hd3a* expression in LD. Weak expression of *Ghd7* in SDs allows induction of the *Ehd1* gene, leading to activation of *Hd3a* expression (Fig. 2) (Itoh *et* *al*., 2010).

### Photoperiod‐induced flowering through key integrators

SD conditions accelerate heading by promoting expression of *Hd3a* in a manner dependent on *Hd1* and *Ehd1*. Expression of *Ehd1* can be upregulated by Days to heading 3 (DTH3, also known as OsSOC1, OsMADS50) (Lee *et* *al*., 2004; Bian *et* *al*., 2011), *Ehd2* (Matsubara *et* *al*., 2008; Wu *et* *al*., 2008), *Ehd3* (Matsubara *et* *al*., 2011), *Ehd4* (Gao *et* *al*., 2013), and *OsMADS51* (Kim *et* *al*., 2007), and downregulated by *Ghd7* (Xue *et* *al*., 2008), Days to heading 8 (DTH8, also known as Ghd8) (Wei *et* *al*., 2010; Yan *et* *al*., 2011), OsCONSTANS‐like 4 (OsCOL4) (Lee *et* *al*., 2010), OsCONSTANS‐like 10 (OsCOL10) (Tan *et* *al*., 2016), Heme activator protein like 1 (OsHAPL1) (Zhu *et* *al*., 2017), and Delayed heading date1 (DHD1) (Z. Y. Zhang *et* *al*., 2019). LD conditions suppress heading, and the expression level of Hd3a is much lower. Under LD conditions, RFT1 also functions as a florigen. Most LD heading date genes regulate florigen gene expression through Ehd1. For example, DTH3, OsMADS51, OsMADS56, Ehd2, Ehd4 and SIP1 (SDG723 Interaction Protein 1) induce Ehd1 expression, whereas Ghd7, DTH8, DTH7, OsCOL4, OsCOL10, OsLFL1 (Rice LEAFY COTYLEDON 2 and FUSCA 3‐LIKE 1), OsBBX14 (Rice B‐box 14), OsABF1 (ABA responsive element binding factor 1), HBF1 (Hd3a BINDING REPRESSOR FACTOR 1), RE1 (Regulator of Ehd1) and RIP1 (OsRE1‐INTERACTING PROTEIN 1) repress Ehd1 expression. Among them, proteins encoded by HBF1, SIP1 and RE1 can directly bind to the promoter of Ehd1 (Brambilla *et* *al*., 2017; Jiang *et al*., 2018, 2018,2018, 2018; Chai *et al*., 2020). However, transcriptional and genetic evidence revealed that DTH2 (Days to heading on chromosome 2) is likely to regulate florigen expression independently of Ehd1 (Wu *et* *al*., 2013; Yokoo *et* *al*., 2014).

### Floral activation in the SAM by the florigen activation complex

The florigen proteins Hd3a and RFT1 are transported to the SAM after induction in leaves. In the SAM, they form the florigen activation complex (FAC) with bZIP transcription factor OsFD1 and 14‐3‐3 protein of the Gf14 family to induce expression of the primary targets, including members of MADS‐box transcription factor family genes that control the phase transition of SAM from vegetative to reproductive development (Taoka *et* *al*., 2011). Both yeast‐two‐hybrid assays and transgenic studies demonstrated that phosphorylation of OsFD1 is important for binding to 14‐3‐3 protein and is a rate‐limiting step for FAC formation (Taoka *et* *al*., 2011). FACs can also form in rice leaves, and these complexes are required to activate a positive feedback loop on *Ehd1*, *Hd3a* and *RFT1* expression. In addition, two additional bZIPs, including Hd3a BINDING REPRESSOR FACTOR1 (HBF1) and HBF2, form alternative FACs with the florigens and reduce *Hd3a* and *RFT1* expression to delay flowering. This situation suggests that Hd3a and RFT1 can regulate their own expression in leaves through forming antagonistic transcription factor complexes to fine tune photoperiod‐induced flowering responses (Brambilla *et* *al*., 2017). In addition, *RICE* *CENTRORADIALIS* (*RCN*) genes encoding PEBP‐like proteins are homologous to the Arabidopsis TERMINAL FLOWER 1 (TFL1) and have similarity to the florigens. RCN represses flowering through competition with Hd3a for 14‐3‐3 binding, and further combines with OsFD1 to form a FAC‐like hexameric complex named the florigen repression complex (FRC). The balance between FAC and FRC modulates florigen activity to optimise inflorescence development (Kaneko‐Suzuki *et* *al*., 2018). We recently identified a new CONSTANS‐like CCT domain protein called Delay heading date 4 (DHD4), which also represses flowering by affecting formation of the FAC complex in the SAM. By contrast with RCNs that interact with OsFD1 mediated by 14‐3‐3, DHD4 directly interacts with OsFD1 and affects the formation of FAC (Cai *et* *al*., 2020), and possibly represents another type of flowering regulation mechanism in SAM.

## Transcriptional and post‐transcriptional regulation of key heading date regulators

### Dual functional regulation of Hd1

Hd1 is the sole homologue of Arabidopsis CO. CO is generally known as a strong LD‐specific floral promoting factor in Arabidopsis (Putterill *et* *al*., 1995), although it may also play a role in repressing flowering under SD conditions (Luccioni *et* *al*., 2019). However, the function of Hd1 has diverged during evolution. It advances heading by activating transcription of the florigen genes under SDs, but delays heading under LD conditions (Yano *et* *al*., 2000). Post‐transcriptional regulation of Hd1 must also be involved in the functional conversion and other aspects of Hd1 function because *Hd1* has a similar expression pattern in different photoperiods (Izawa *et* *al*., 2002; Ishikawa *et* *al*., 2011).

Whether Hd1 functions as an activator or suppressor of heading depends on the red‐light photoreceptor phytochrome B (PhyB). Mutation of *PhyB* or phytochrome chromophore synthesis gene *photoperiod* *sensitivity* *5* (*Se5*) attenuates the reversible function of Hd1 in which Hd1 works as an activator under all photoperiods in the absence of phytochromes. As *Hd1* expression is not affected in the *se5* mutant it is thought that phytochromes may affect Hd1 function post‐transcriptionally (Izawa *et* *al*., 2002).

Hd1 also loses LD heading suppression function and consistently promotes flowering under SD and LD in *Ghd7*‐ or *DTH8*‐deficient backgrounds (Nemoto *et* *al*., 2016; Du *et* *al*., 2017; Zhang *et* *al*., 2017; Zong *et* *al*., 2021). Transcriptional analyses revealed that *Ghd7* and *Hd1* do not regulate each other’s transcription levels, suggesting that the observed genetic interaction might occur post‐transcriptionally (Nemoto *et* *al*., 2016). Hd1 was shown to interact with flowering repressor Ghd7, and the Hd1–Ghd7 complex binds to specific sites in the *Ehd1* promoter and suppresses expression of *Ehd1* and florigen genes during daylight hours under LD conditions (Nemoto *et* *al*., 2016). As the interaction occurs between the transcription‐activating domain of Hd1 and CCT domain of Ghd7, it was hypothesised that the interaction might block or weaken the transcriptional activation activity of Hd1 and release the downstream gene transcriptional repression activity of Ghd7 (Zhang *et* *al*., 2017). Because the expression level of Ghd7 is significantly lower in SDs (Xue *et* *al*., 2008), less Hd1–Ghd7 complex forms and so Hd1 still promotes heading date on a Ghd7 background under SDs (Nemoto *et* *al*., 2016; Zhang *et* *al*., 2017).

*DTH8* is another key floral repressor under LD conditions (Wei *et* *al*., 2010a,b; Yan *et* *al*., 2011; Dai *et* *al*., 2012). Like Ghd7, DTH8 interacts with Hd1 (Du *et* *al*., 2017; Zhu *et* *al*., 2017). The DTH8–Hd1 complex is necessary for the floral inhibition function of Hd1 in LDs. In *dth8* background, Hd1 loses its alternative function and acts as an activator of *Hd3a* expression to promote flowering both in SD and LD conditions in the 93‐11 background. Another report suggested that DTH8 alone could not convert Hd1 function, but enhanced the effect of Ghd7 on the Hd1 functional conversion in the ZS97 background (Z. Y. Zhang *et* *al*., 2019). The exact mechanism by which DTH8 affects the Hd1 functional switch is not clear, but one possibility is that it acts by changing H3K27me3 levels in the *Hd3a* promoter (Du *et* *al*., 2017).

Several recent studies have revealed that both Ghd7 and DTH8 can interact with Hd1 and that DTH8 also interacts with Ghd7. Therefore, although *Hd1* has the primary function for promoting expression of *Hd3a/RFT1* and flowering regardless of day length, it can form a ternary repressive complex with DTH8 and Ghd7 to mediate photoperiodic heading (Cai *et* *al*., 2019; Zong *et* *al*., 2021). This view is supported by the observation that a functional *Ghd7*, *DTH8* and *Hd1* combination showed significantly stronger photoperiod sensitivity than other combinations in both near‐isogenic lines and natural populations (Zhang *et* *al*., 2015; Wang *et* *al*., 2019; Z.Y. Zhang *et* *al*., 2019; Zong *et* *al*., 2021), although there might be other types of transcriptional or post‐transcriptional regulation involving one or more of them (Wang *et* *al*., 2019). For example, Hd1, DTH8 and distinct NF‐YC subunits reported to form a trimeric complex that bound to the *CO* *Response* *Element* *2* (*CORE2*) in the *Hd3a* promoter, and Days to heading 7 (DTH7, also known as Hd2, OsPRR37 or Ghd7.1, another CCT domain‐containing protein strongly repressing flowering) can replace Hd1 in the complex (Goretti *et* *al*., 2017). Hd1 also interacts with DTH8 and OsHAPL1 and is likely to form a complex with general transcription factors to regulate the transcription of target genes to control heading date in rice (Zhu *et* *al*., 2017). These results indicated that Hd1, Ghd7, DTH8 and DTH7 do not act independently, but assemble into complexes to regulate the rice photoperiodic pathway.

Hd1 also interacts with Rice Halotolerance Protein 3 (OsHAL3), a flavin mononucleotide‐binding protein reported as a blue‐light sensor that does not contain a DNA‐binding domain. The interaction is inhibited by white or blue light. The OsHAL3–Hd1 complex promotes heading under SD, and activates *Hd3a* expression by direct binding to the *Hd3a* promoter. *OsHAL3* expression exhibits a diurnal pattern that peaks at 4 h before light onset under SD, but peaks 4 h after light onset under LD conditions. It was proposed that the more *OsHAL3* coincides with the midnight‐expressed *Hd1* under SD, more OsHAL3–Hd1 complex is formed to activate *Hd3a* expression. The level of expression of OsHAL3 under LDs peaks during daytime, and light inhibits the formation of the OsHAL3–Hd1 complex. There is no obvious induction of *Hd3a* mRNA levels by OsHAL3 (Su *et* *al*., 2016).

Heading date associated factor 1 (HAF1), a C3HC4 RING domain‐containing E3 ubiquitin ligase, was identified as an Hd1 interacting protein in yeast‐two‐hybrid assays. HAF1 mediates ubiquitination and regulates circadian accumulation of Hd1 by targeting it for degradation via the 26S proteasome‐dependent pathway. The *haf1* *hd1* double mutant flowers as late as *hd1* plants only in SD conditions, suggesting that HAF1 is essential for regulating heading by modulation of the diurnal rhythm of Hd1 protein in SDs (Yang *et* *al*., 2015). Additional research revealed that HAF1 also mediates ubiquitination and modulates the diurnal rhythm of OsELF3 (an orthologue of the Arabidopsis ELF3, also known as Hd17) under LDs (Zhu *et* *al*., 2018).

### Protein level regulation of key floral integrator Ehd1

*Ehd1* encodes a B‐type response regulator that functions upstream of florigen genes and promotes heading under both SD and LD conditions. *Ehd1* integrates various upstream flowering signals and is recognised as key floral inducer in rice (Doi *et* *al*., 2004; Shrestha *et* *al*., 2014). In addition to regulation of the transcription level, post‐translational modifications also play critical roles in Ehd1 function. It has been reported that the activity of Ehd1 protein in promoting *Hd3a* and *RFT1* expression is repressed by *OsPhyA* in a day length‐dependent manner (Osugi *et* *al*., 2011). Ehd1 activity can also be regulated by phosphorylation modification, as mutation of the key amino acid Asp‐63 to Glu to mimic a constitutive phosphorylated state greatly enhanced its function and caused extremely early heading. A separate study revealed that phosphorylation of Asp‐63 is required for formation of Ehd1 complexes, and dimerisation is important for normal Ehd1 function (Cho *et* *al*., 2016). Interestingly, OsRR1, a type‐A response regulator, can inhibit Ehd1 activity by direct interaction with Ehd1 to form an inactive heterodimer, and delays heading (Cho *et* *al*., 2016).

### Post‐translational regulation of Ghd7 function

Previous studies have found that *Ghd7* transcription was increased or unaltered in *phyB* mutants (Osugi *et* *al*., 2011; Weng *et* *al*., 2014). However, the level of Ghd7 protein was significantly decreased in a *phyB* mutant under LD conditions, suggesting that *PhyB* maintains the level of *Ghd7* protein (Weng *et* *al*., 2014). More recently, one study found that constitutive expression of *Ghd7* on a *se5* background failed to accumulate Ghd7 protein or delay heading, suggesting that phytochromes are necessary for Ghd7 protein stability and normal function (Zheng *et* *al*., 2019). Further *in* *vitro* and *in* *vivo* analyses revealed that phytochromes (mainly OsPHYA and OsPHYB) directly interact with Ghd7 and stabilise Ghd7 protein. OsGI also interacts with Ghd7 and mediates its degradation in a 26S proteasome‐dependent manner. Interestingly, both OsPHYA and OsPHYB inhibit the interaction between OsGI and Ghd7, therefore blocking OsGI‐mediated Ghd7 degradation (Zheng *et* *al*., 2019). In addition to regulation of protein stability by phytochromes and OsGI, Ghd7 was also reported to be phosphorylated by Heading date 16 (Hd16), a casein kinase‐I protein (Hori *et* *al*., 2013). Hd16 delays heading by interaction with, and phosphorylation of, Ghd7, thereby enhancing the function of Ghd7, and suppressing expression of *Ehd1* and downstream florigen genes (Hori *et* *al*., 2013).

### Fine‐tuning photoperiod‐controlled flowering in rice by phosphorylation of floral regulators

In addition to the above‐mentioned phosphorylation and enhancement functions of Ghd7, Hd16 was earlier identified as EARLY FLOWERING 1 (EL1), which phosphorylates DELLA protein SLR1 (Slender rice 1, a key component in gibberellin signalling). Phosphorylation of SLR1 is important for maintaining its stability and negative regulation of gibberellin signalling (Dai & Xue, 2010). As phosphorylation of both Ghd7 and SLR1 results in suppression of heading under LD conditions, it is possible that Hd16 regulates both photoperiod and gibberellin responses by targeting different substrates in the rice flowering pathway. DTH7/OsPRR37/Ghd7.1/Hd2 is another substrate of Hd16. DTH7 directly interacts with, and is phosphorylated by, Hd16 in the middle and CCT domain‐containing C‐terminal regions (Choon‐Tak *et* *al*., 2015). DTH7 can also be phosphorylated in the middle region by casein kinase 2α (CK2α) encoded by the *Heading* *date* *6* (*Hd6*) gene (Choon‐Tak *et* *al*., 2015). Hd6, identified by QTL mapping, delays heading specifically under LD conditions. Other genetic studies have revealed that *Hd6* delays heading only when *Hd1* is functional. Extensive studies have revealed that *Hd6* does not regulate *Hd1* transcription, but represses expression of *Hd3a* and *RFT1* in an *Hd1*‐dependent manner, suggesting that *Hd6* modulates *Hd1* function post‐translationally. However, Hd6 does not directly phosphorylate Hd1 protein, implying the presence of an unknown Hd6 target that might function with Hd1 (Ogiso *et* *al*., 2010). It has been reported that *Hd1* acts genetically downstream of *DTH7* to delay flowering time in LD conditions (Lin *et* *al*., 2000), and that *DTH7* is necessary for LD heading repression function for conversion of *Hd1* (Z. Y. Zhang *et* *al*., 2019). Given the direct phosphorylation of DTH7 by Hd6 (Choon‐Tak *et* *al*., 2015), *DTH7* is likely to be a candidate protein that bridges the gap between *Hd6* and *Hd1*. Further genetic and biochemical evidence is necessary to confirm this hypothesis.

Hd1 was also reported to be phosphorylated by OsK4, another protein kinase identified as Heading Date Repressor1 (HDR1) interaction protein. The HDR1–OsK4 complex acts to inhibit flowering in LDs by both transcriptional regulation of *Hd1*/*Ehd1* expression, and probably by regulation of the phosphorylation state of Hd1 (Sun *et* *al*., 2016). Hd6 can phosphorylate OsLHY, an orthologue of the Arabidopsis circadian oscillator components LATE ELONGATED HYPOCOTYLN(LHY) (Ogiso *et* *al*., 2010), but mutation in either *Hd6* or *Hd16* does not affect the circadian clock (Nemoto *et* *al*., 2018). Natural variations in *Hd6* and *Hd16* are involved in fine tuning the critical day length in photoperiod‐controlled heading and may contribute to further northward expansion of rice cultivation areas (Nemoto *et* *al*., 2018).

## Domestication and genetic improvement promote expansion of the rice growing region

The ancestral species of Asian cultivated rice (*Oryza* *rufipogon* Griff.) is SD sensitive and its distribution is limited to low‐latitude tropical and subtropical regions. After natural selection and artificial domestication, cultivated rice is now distributed across an extremely wide range of latitudes from 53°N to 40°S, and is grown under both LD and SD conditions (Koo *et* *al*., 2013). Weakened photoperiod sensitivity is a critical factor for adaptation of rice to high‐latitude regions. Allelic variants of several heading date genes have contribute to the northward advance of rice growing in Asia. *Hd1*, *Ghd7*, *DTH8*, *Hd16*, *DTH7* and *PhyB* are flowering repressor genes under LDs. Combined *Hd1*, *Ghd7* and *DTH8* alleles produce strong photoperiod sensitivity, and are preferably distributed to low latitudes (Zong *et* *al*., 2021). Deletion or weakened alleles of these genes are associated with early flowering under natural day length and are preferably distributed to high latitudes (Xue *et* *al*., 2008; Takahashi *et* *al*., 2009; Wei *et al*., 2010; Huang *et al*., 2012; Koo *et al*., 2013; Gao *et al*., 2014; Kwon *et al*., 2014). Combinations of weak alleles of *Ghd7*, *DTH8* and *DTH7* act additively to reduce photoperiod sensitivity, and are likely to occur in high‐latitude areas such as the north‐eastern provinces of China (Gao *et al*., 2014). Further analysis revealed that genetic variations in *DTH8*, *Ghd7*, *Hd1*, *DTH7*, *PhyB* and *OsCOL4* are correlated with differences in heading date, and a minimum combination of four weak alleles is required for the successful cultivation of rice at latitudes above 30°N (Zheng *et al*., 2016; Cui *et al*., 2020). Moreover, flowering activation genes, such as *DTH2*, *Ehd4* and *RFT1*, have also undergone intensive selection and contributed to regional adaptation of rice to higher latitudes (Gao *et al*., 2013; Wu *et al*., 2013; Zhao *et al*., 2015).

## Heading date genes contribute to heterosis in rice

Studies on natural variation and various genetic populations have revealed that several major heading date genes were also important heterosis genes, such as *Hd3a*, *DTH8* and *Ghd7* (Huang *et al*., 2016; Li *et al*., 2016). The heterozygous state of *Hd3a* is common in the most planted hybrid rice cultivars grown in China. Among 1063 three‐line hybrids studied, 98.5% of restorer lines had the ancestral type of homozygous *Hd3a* allele (*Hd3a*/*Hd3a*), whereas 76.7% of cytoplasmic male sterile lines were homozygous *hd3a* allele (*hd3a*/*hd3a*). The heterozygous state (Hd3a/hd3a) had higher grain numbers per panicle, seed setting rate and grain yield per plant than their parents (Huang *et al*., 2016). The heterozygous state of the tomato florigen *SINGLE* *FLOWER* *TRUSS* (orthologue of rice Hd3a) gene increased the yield in distinct genetic backgrounds and environments (Krieger *et* *al*., 2010). *DTH8* is another major heading date gene that contributes to yield heterosis in hybrid rice cultivars. The heterozygous state, consisting of a strong allele and a nonfunctional allele of *DTH8* (*DTH8*/*dth8*), is present in many Chinese hybrids, particularly in the two‐line hybrids (40.8%), including the well known superhybrid rice Liang‐you‐pei 9 (LYP9) (Li *et* *al*., 2016). More recently, studies have shown that the *DTH8* locus has been divergently selected among rice subpopulations, and the genetic segment carrying *japonica* type *DTH8* was introgressed into the male parent in modern *indica* hybrid cultivar breeding, and contributed to heterosis with overdominance effects (Huang *et* *al*., 2016; Lin *et* *al*., 2020). Similarly, *Ghd7* has been divergently selected in the male and female parents of hybrid rice and that heterozygous state of *Ghd7* consisting of a strong allele and a weak or nonfunctional allele was also found to be common in Chinese hybrid rice cultivars (Li *et* *al*., 2016; Lin *et* *al*., 2020).

The underlying mechanism of heterozygosity in heading date genes leading to heterosis is still not clear. One possible explanation is the pleiotropic effect of these heading date genes, for example *Hd3a* promotes lateral branching in addition to inducing flowering (Tsuji *et* *al*., 2015), and *DTH8* and *Ghd7* affect heading date, plant height and grain number per panicle simultaneously (Xue *et* *al*., 2008; Wei *et* *al*., 2010). We postulate that the heterozygous genotype may help to reach a balance on proper flowering time and ideal plant architecture. Whether other heading date genes also contribute to heterosis is an interesting subject, more studies are needed to reveal the underlying mechanism.

## Conclusions and perspectives

Many genes controlling heading date in rice have been identified in recent decades. Our understanding of how these genes coordinately regulate heading date remain fragmentary. Gene expression can be regulated at the transcriptional and post‐transcriptional levels. In addition, there is evidence that epigenetic regulation also plays important roles in flowering time regulation. For example, many histone methylation‐related proteins (such as SDG701, SDG708, SDG724, SDG725, SDG711, SDG718, SDG723, LC2/OsVIL3, OsVIL2 and OsEMF2b) regulate flowering by methylation on the lysine residues in the N‐tails of histone H3 and cause either transcriptional silencing or activation of major flowering genes (Sun *et* *al*., 2012; Sui *et* *al*., 2013; Wang *et* *al*., 2013; Yang *et* *al*., 2013; Choi *et* *al*., 2014; Liu *et* *al*., 2014; Liu *et* *al*., 2016; Liu *et* *al*., 2017; Jiang *et* *al*., 2018; Liu *et* *al*., 2019). *Se14* encodes a Jumonji C‐domain‐containing histone demethylase, *HDT701* (also known as *OsHDT1*) and *OsSFL1* encode components of histone deacetylase, which also strongly affect heading date (Li *et* *al*., 2011; Yokoo *et* *al*., 2014; Cho *et* *al*., 2018; Geng *et* *al*., 2020). Interested readers on this subject are referred to previous reviews (Shi *et* *al*., 2014; Sun *et* *al*., 2014).

Inspired by the well established flowering regulation pathways in the LD plant Arabidopsis, and through analysis of the expression of heading date genes in different rice genetic backgrounds, an evolutionary conserved *OsGI*–*Hd1*–*Hd3a* pathway and a rice‐specific *Ehd1* pathway were proposed. *Ehd1* was earlier proposed to function independently of *Hd1*, as it can effectively promote heading in a *Hd1*‐deficient background (Doi *et* *al*., 2004). However, some studies at the protein level have uncovered physical interactions between Hd1 and Ghd7, which form a complex that binds to the *Ehd1* promoter to repress its expression (Nemoto *et* *al*., 2016), suggesting that day length responses in rice are controlled by integration of the two pathways into Ehd1 to repress heading under LD conditions.

### Future perspective

Compared with the transcriptional regulation pathway in heading date, our knowledge on regulation of protein levels is still limited. Post‐transcriptional regulation of flowering has been extensively studied in Arabidopsis. Although there is conservation of the flowering mechanism between rice and Arabidopsis, rice is a SD plant and it is inappropriate to directly transfer knowledge from a LD plant such as Arabidopsis to rice. Further study at the level of protein regulation of heading date in rice will be helpful to distinguish the functions and evolutionary processes in photoperiod‐controlled growth of plants. Taking advantage of rapidly improving *in* *planta* protein–protein interaction detection methods, including traditional affinity purification‐mass spectrometry (AP‐MS) and newly developed proximity labelling methods such as TurboID‐mediated proximity labelling (Branon *et* *al*., 2018), large‐scale identification of interacting proteins with known heading date regulators is now possible. CRISPR (clustered regularly interspaced short palindromic repeats)‐based genomic editing will also facilitate the rapid unveiling of the functions of interacting proteins. Moreover, a recently proposed initiative to tag all rice proteins *in* *locus* using a CRISPR‐Cas‐based targeted insertion method (named the Rice Protein Tagging Project, RPTP) should greatly facilitate functional studies on heading date and other biological processes in rice (Lu *et* *al*., 2020).

Another important limitation of the study of heading date in rice is the diverse genetic backgrounds used in different studies, by contrast with the relatively centralised background in Arabidopsis (such as the Col‐0 ecotype). As functions of genes can vary in different backgrounds it is often difficult to integrate some conflicting results from multiple studies. We propose that the rice community systematically generate genetic resources in a common genetic background (such as Nipponbare for *japonica* and 93‐11 for *indica*) through genomic editing, transgenesis and traditional backcrossing. Integrating different genes and building a complete heading date pathway with the support of genetic evidence in a single background will advance our understanding of the plant flowering mechanism more rapidly, especially at the protein level.

### Applications in rice breeding

**Fig. 3:**
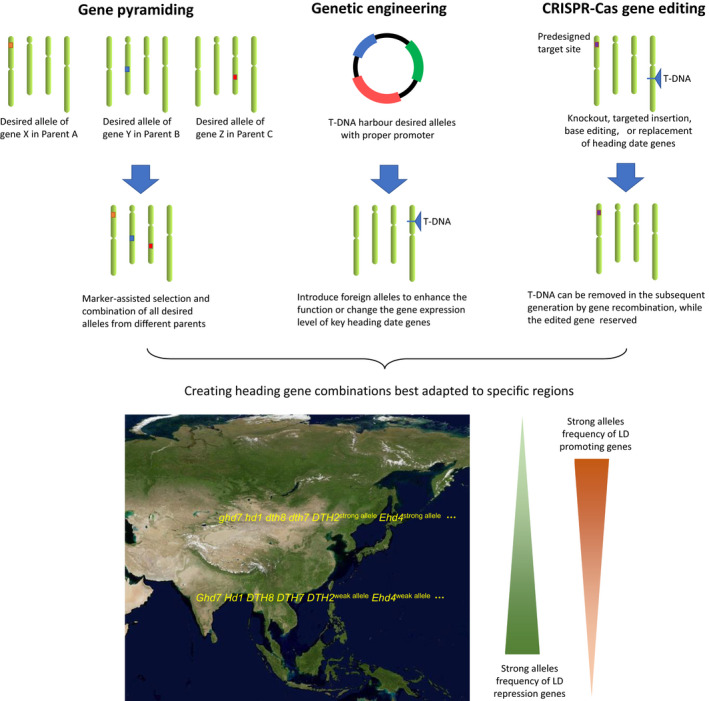
Strategies for breeding rice cultivars with optimum heading dates. Rational design of long day (LD) repression genes (such as *Ghd7* and *DTH8*) and LD promoting genes (such as *DTH2* and *Ehd4*) for rice cultivars adapting to different latitudes. Three strategies can be used, including pyramiding heading date alleles by traditional cross‐breeding with the assistance of molecular markers, modulating the function or expression level of heading date genes by genetic engineering, and modifying heading genes by CRISPR‐Cas gene editing technology.

The breeding of rice cultivars with optimum heading dates is crucial for high yield in specific cropping areas. In traditional breeding of rice (a predominantly self‐pollinated species) we select individuals with the desired heading date. However, this work becomes challenging in hybrid rice breeding, as testing heading date of every hybrid combination is labour and time consuming. This limitation is especially a problem in *indica* × *japonica* hybrids in which delayed heading is common. This bottleneck of unpredictable heading date limits the exploitation of substantial intersubspecies heterosis. Over past decades, many genes controlling flowering in rice have been cloned and functionally analysed, and their allelic distributions in different cropping areas have also been characterised. This should allow the rational design of cultivars with heading gene combinations best adapted to specific environmental conditions (Fig. 3). One approach is to pyramid heading date alleles in predetermined combinations by traditional cross‐breeding with the assistance of molecular markers, as attempted in a previous study (Wei *et* *al*., 2010). The second approach is to modulate the function or expression levels of heading date genes by genetic engineering, as many genes regulate rice heading date quantitatively. This can be achieved through the downregulation of genes by RNA interference or upregulation of genes by specific promoters (Hu *et* *al*., 2019). The third approach is to modify heading date genes using gene editing technology. CRISPR‐Cas genome editing enables targeted modification of specific heading date genes without affecting the genetic background, including targeted gene knockout, nucleotide base editing, insertion, deletion or replacement (Zhu *et* *al*., 2020). Moreover, multiple targets can be changed simultaneously, making breeding of new varieties with predetermined heading date gene combinations more efficient. Although exogenous T‐DNA is usually needed to deliver CRISPR‐Cas reagents into rice cells, these genetic elements can be easily removed in later generations to obtain transgene‐free edited lines. These advantages make the CRISPR‐Cas approach a fast, feasible and reliable technology in regulating heading date in rice.

## Author contributions

JMW conceived the article. SRZ and SSZ contributed to the development of the ideas expressed within it. SRZ wrote the manuscript, with contributions from SSZ, SC, HGH, HQW, BYH, LC, ZX, LLL, LJ and HYW. SRZ and SSZ contributed equally to this work.

## Data Availability

The data that support the findings of this study are available from the corresponding author upon reasonable request.
